# α7 nicotinic acetylcholine receptor agonist GTS-21 attenuates DSS-induced intestinal colitis by improving intestinal mucosal barrier function

**DOI:** 10.1186/s10020-022-00485-6

**Published:** 2022-06-03

**Authors:** Ziping Ye, Yunjuan Zhu, Nana Tang, Xiaojing Zhao, Jingyue Jiang, Jingjing Ma, Hongjie Zhang

**Affiliations:** 1 Department of Gastroenterology, The First Affiliated Hospital of Nanjing Medical University, 300 Guangzhou Road, Nanjing, 210029 Jiangsu Province People’s Republic of China; 2 Department of Gastroenterology, Ganyu District People’s Hospital of Lianyungang City, Lianyungang, Jiangsu Province People’s Republic of China

**Keywords:** α7 nicotinic acetylcholine receptor, GTS-21, Colitis, Intestinal mucosal barrier function

## Abstract

**Background and aims:**

Cholinergic output, which could modulate innate immune responses through stimulation of α7 nicotinic acetylcholine receptor (α7nAChR), might be a target to minimize tissue damage in autoimmune disease. GTS-21, a selective α7nAChR agonist, has previously demonstrated to inhibit synovium inflammation in rheumatoid arthritis. In this study, we investigated the effect of GTS-21 on dextran sulfate sodium (DSS)-induced colitis model and its potential mechanism.

**Methods:**

Male BABL/c mice (n = 32) were randomly divided into four groups: normal control group, DSS-induced colitis group, GTS-21 treatment with or without α7nAChR antagonist α-BGT treatment group. Disease activity index (DAI), histological activity index (HAI) and colonic macroscopic damage were evaluated. Fluorescein isothiocyanate (FITC)–dextran assay was applied to measure intestinal permeability. The expressions of tight junction (TJ) proteins and NF-κB associated proteins were detected by Western blot.

**Results:**

GTS-21 could decrease DAI scores, HAI scores, intestinal permeability and reduce the intestinal bacterial translocation in DSS-induced colitis group, whereas α7nAChR antagonist α-BGT could impair this protective influence. The expressions of TJ proteins were increased with administration of GTS-21 both in vivo and in vitro. Furthermore, GTS-21 also inhibited the NF-қB activation in intestinal epithelial cells and colitis model, while α-BGT reversed the inhibitory effect.

**Conclusion:**

The α7nAChR agonist GTS-21 attenuated DSS-induced colitis through increasing expressions of TJ proteins in colon tissues and improved intestinal barrier function, which might be due to  modulating NF-қB activation in intestinal epithelial cells.

**Supplementary Information:**

The online version contains supplementary material available at 10.1186/s10020-022-00485-6.

## Introduction

Inflammatory bowel disease (IBD) is a chronic inflammatory digestive tract disease including two major forms: Crohn’s disease (CD) and ulcerative colitis (UC). The etiology and pathogenesis of IBD were still incompletely clear. Some studies demonstrated that autonomic nerve imbalance might  contribute to the inflammatory progression in colitis models (O’Mahony et al. 2009, Abraham and Medzhitov 2011; Bonaz et al. 2021). Inflammatory cytokines could be modulated by neurotransmitters including acetylcholine (Ach) (Rueda Ruzafa et al. 2021, Zheng et al. 2021). The impaired parasympathetic function was found in up to 35% UC patients and 48% of CD patients (Lindgren et al. 1993; Straub et al. 1997). Vagotomised mice developed more severe colitis with increased   phosphorylated NF-κB  and pro-inflammatory cytokines (Gowayed et al. 2019). Vagus nerve stimulation could inhibit production of proinflammatory cytokines and modulate balance between different immune cells in colon tissue (de Araujo and de Lartigue 2020), which was called cholinergic anti-inflammatory pathway (CAP) (Tracey 2002).

The CAP has been reported to efficiently reduce loss of barrier function and intestinal damage (Okumura et al. 2020). When intestinal barrier function was impaired, the pathogens in luminal could penetrate into tissue (Alipour et al. 2016, Drolia et al. 2018). A key component of the intestinal barrier, which was composed of tight junctions (TJs), adherens junctions, desmosomes and gap junctions (Farquhar and Palade 1963), was the intercellular junction complexes between adjacent intestinal epithelial cells. The increase in intestinal TJ permeability induced by proinflammatory cytokines allowed paracellular permeation of harmful luminal agents and promoted inflammation (Li et al. 2010; Chelakkot et al. 2018). The CAP inhibited inflammation via stimulation of α7-nicotinic acetylcholine receptor (α7nAChR) (Bencherif et al. 2011; Matteoli and Boeckxstaens 2013). α7nAChR, which was identified as the cholinergic receptor and mediated the anti-inflammatory effect (van Der Zanden et al. 2009, Pavlov and Tracey 2012), was found in macrophage, lymphocyte and epithelial cells (Gahring and Rogers 2006; Zanetti et al. 2016). Compared with wild-type mice, DSS-induced colitis was more severe in α7nAChR KO mice (Wang et al. 2003). The α7nAChR agonist choline chloride ameliorated colitis (Salaga et al. 2016).

GTS-21, a selective α7nAChR agonist, could lower the expressions of serum tumor necrosis factor (TNF) and high-mobility group box1 (HMGB1) in mice with lethal endotoxemia, sepsis and collagen-induced arthritis (Wang et al. 2003, 2004; Pavlov et al. 2007). GTS-21 also had a protective effect in rheumatoid arthritis through CAP (Wu et al. 2014). Does GTS-21 also have a protective effect on colitis? The aim of this study was to determine the effects of GTS-21 on intestinal damage, intestinal permeability, intestinal mucosal barrier function and the possible molecular mechanism. In this study, we found that GTS-21 significantly improved intestinal mucosal barrier function by increasing the expressions of TJ proteins and attenuating intestinal permeability through α7nAChR, which might contribute to ameliorating DSS-induced colitis.

## Materials and methods

### Animals and experimental procedure

Male BALB/c mice (24–30 g) were purchased from CAVENS animal center (Chang Zhou, China, SCXK (SU) 2011-0003). The mice were housed in the specific pathogen-free (SPF) facility at Nanjing University. All protocols were approved by the Nanjing University of Science and Technology Animal Care and Use Committee. In addition, the animals received humane care in compliance with the Principles of Laboratory Animal Care.

BALB/c mice were randomly divided into four groups (8 mice/each group): Group I (Control, Ctrl): mice drunk sterile tap water freely and were intraperitoneally injected with saline daily from day 0 to day 7; Group II (DSS group): mice received 3.5% DSS (molecular weight 36–50 kDa, MP Biomedicals, USA) in drinking water freely from day 1 to day 7 and were intraperitoneally injected daily with saline from day 0 to day 7; Group III (GTS-21 group): mice were intraperitoneally injected daily with GST-21 (10 mg/kg/day, sigma, USA) from day 0 to day 7 and received 3.5% DSS in drinking water freely from day 1 to day 7; Group IV (α-BGT group): mice were pre-treated with α-BGT (0.1 mg/kg/day, abcam, USA) via intraperitoneal injection 30 min prior to GTS-21 injection from day 0 to day 7 and received 3.5% DSS in drinking water freely from day 1 to day 7. Animals were euthanized on day 8.

### Cell culture and GTS-21 intervene experiments

Caco2 cells were purchased from China Cell Culture Center (Shanghai, China). Dulbecco’s modified Eagle’s medium (DMEM; Gibco, USA) with 10% fetal bovine serum (FBS, Gibco) and 1% penicillin and streptomycin was used for growth of Caco2 cells. Caco2 cells were given different treatment as follows: ① The different concentrations of TNF-α (0, 0.1, 1, 10, 25, 50 ng/ml) (peprotech, USA) were given. Finally, 25 ng/ml was chosen as the optimal concentration of TNF-α for treating Caco2 cells; ② GTS-21 (100 ng/ml) were given for 30 min prior to TNF-α; ③ α-BGT (50 ng/ml) were pre-treated for 30 min prior to GTS-21, then 30 min later treated with TNF-α.

### Assessment of severity of colitis

To evaluate the severity of colitis(Kim and Berstad 1992), all mice were estimated daily for disease activity index (DAI) scores, including weight loss, stool consistency, occult/gross bleeding as described previously(Kihara et al. 2003). The histology activity index (HAI) was used to grade the severity of intestinal inflammation in accordance with a previously publication (Kihara et al. 2003). Mice were sacrificed on day 8 and the left hemi-colon were removed, fixed in 4% paraformaldehyde.

### Assessment of intestinal permeability

Intestinal barrier function was assessed by intestinal permeability assay (Yasuda et al. 2006; Li et al. 2011). After mice were killed, a midline laparotomy incision was performed. 5 cm of the terminal ileum and the right colon were removed and washed gently, then one side of the intestine was ligated. A solution 100 ml of 40 mg/ml 4-KDa FITC-Dextran (Sigma, St. Louis, MO) was applied into the intestinal lumen, and then another side was ligated. The intestinal pouch was immersed gently in 10 ml of PBS at 37 °C for 60 min. The intestinal wall permeability was evaluated by measuring the leaked amount of FITC-dextran outside the intestinal pouch. Fluorescence was measured in three replicate wells for each sample (200 μL) in black 96-well microtiter plates (Proxiplate-96 F, Perkin Elmer) using a Victor TM X4 Plate reader (Perkin Elmer) with excitation at 485 nm and emission at 535 nm. Concentrations were calculated from a standard curve.

### Fluorescence in-situ hybridization (FISH) of EUB338

Bacteria penetrating in DSS-induced colitis was determined by fluorescence in-situ hybridization (FISH) of EUB338 probe as described previously (Alipour et al. 2016). Colon tissues were obtained from left hemi-colon, and were paraffin embedded, cut into slices. FISH probe EUB338 was used to verify all members of the bacteria family (Integrated DNA technologies, genepharma, Shanghai, ON, China). After dewaxed, hydrated, degenerated, the sections were hybridized with 50 μg/ml of EUB338. Images were acquired with an Axio Examiner Z1 LSM 5100 confocal microscope.

### Western blot analysis

Total Caco-2 monolayer cells or mice colon tissues were lysed by using RIPA lysis buffer (Beyotime, China) and quantified by a Pierce BCA protein assay kit. Equal amounts of each extract were electrophoresed on 10% sodium dodecyl sulfate–polyacrylamide gels and transferred to polyvinylidene fluoride membranes. The membranes were blocked for 1 h with 5% dried skim milk in TBST, followed by overnight incubation at 4 °C with primary antibody (ZO-1, 1:500, invitrogen, USA; claudin-1, 1:1000, abcam, USA; JAM-1, 1:1000, abcam, USA; occludin, 1:1000, proteintech, USA; NF-қB p65, 1:1000, CST, USA; phosphorylated NF-қB p65, 1:500, CST, USA; IκBα, 1:1000, CST, USA; phosphorylated IκBα, 1:1000, CST, USA; CHRNA7, 1:1000, Proteintech, China; GAPDH, 1:5000, beyotime, China). The blots were incubated with the corresponding secondary antibodies for 1 h at 37 °C. The bands were detected by enhanced chemiluminescence and normalized to GAPDH expression. Data were quantified by ImageLab2.0.1 software.

### Immunofluorescence microscopy

The subcellular localization of the TJ associated proteins (ZO-1, occludin, claudin-1, JAM-1) both in colon tissues and Caco2 monolayer cells were assessed by immunofluorescence as described previously(Wu et al. 2011; Devriese et al. 2017). The colon tissues sections were de-waxed, hydrated with dimethylbenzene and ethyl alcohol, fixed with stationary liquid (beyotime, China). Cell monolayers in coverslip were fixed with 4% paraformaldehyde. Fixed tissue and cell monolayers were blocked at room temperature for 40 min, followed by incubation with primary antibody (ZO-1, 1:50, invitrogen, USA; claudin-1, 1:100, abcam, USA; JAM-1, 1:100, abcam, USA; occludin, 1:100, proteintech, USA; p65, 1:100, abcam, USA; CHRNA7, 1:100, Proteintech, China) overnight at 4 °C. Sections were then washed and incubated with the corresponding secondary antibodies, stained nucleus with DAPI (1:1000, beyotime, China) for 5 min at room temperature, rinsed with PBS and mounted on the slide using the anti-quenching reagent. The fluorescence was visualized under Axio Examiner Z1 LSM 5100 confocal microscope.

### Statistical analysis

All images were representative of at least three independent experiments. Data were shown as the mean ± standard error of the mean (SEM). Statistical analysis was performed using SPSS version 22.0 (IBM SPSS Statistics, USA) and GraphPad Prism (Prism 5 software, USA). Student’s *t*-test or one-way analysis of variance followed by Tukey’s post-hoc test was used for analysis, *p* < 0.05 (*) was considered significant, *p* < 0.01 (**) was considered highly significant, and *p* < 0.001 (***) was very highly significant.

## Results

### α7nAChR agonist GTS-21 ameliorates DSS-induced colitis

**Fig. 1:**
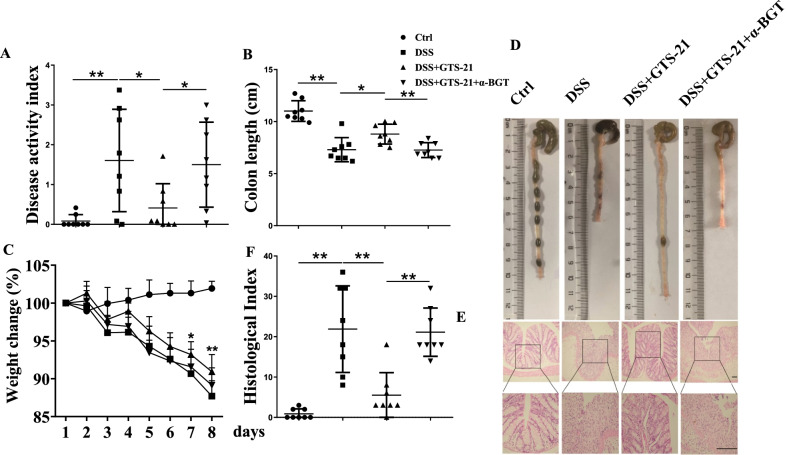
α7nAChR agonist GTS-21 treatment attenuates dextran sodium sulphate (DSS)-induced colitis. **A** Disease activity index (DAI) scores on day 8 was evaluated. *p < 0.05; **p < 0.01. **B** Colon length on day 8. *p < 0.05; **p < 0.01. **C** Weight change (%) from day 0 to day 8. *p < 0.05; **p < 0.01(GTS-21+DSS vs. DSS alone). **D** Gross macroscopic inflammation and length of the large bowel in each group. **E** Hematoxylin and eosin (H&E) was performed on paraffin-embedded distal colon tissue. Magnification: upper panels, ×100; lower panels, ×400. **F** Histopathological Index. **p < 0.01. All data represent as mean ± SEM of 8 mice per group. Scale bar, 500 nm

To evaluate the effects of α7nAChR agonist GTS-21 on the development and severity of experimental colitis, the mice were daily treated with GTS-21. As shown in Fig. 1A, the DAI scores in DSS-induced mice on day 8 were significantly increased compared with control mice, while the DAI scores in colitis mice treated with GTS-21 significantly decreased compared with colitis mice without GTS-21. The mean lengths of colon and the weight of mice were significantly improved in GTS-21 group compared with DSS group (Fig. 1B–D). Furthermore, we evaluated the severity of colitis by histopathological analysis, the villus necrosis, hemorrhage and inflammatory cell infiltration in the lamina propria which were showed in colonic tissues from DSS colitis mice. GTS-21 treatment drastically alleviated inflammatory cell infiltration in colon (Fig. 1E). The HAI in GTS-21 group also decreased compared with that in DSS group (Fig. 1F). These data suggested that GTS-21 could alleviate DSS-induced colitis.

### GTS-21 attenuates intestinal permeability and inhibits intestinal bacterial translocation in DSS-induced colitis mice

**Fig. 2:**
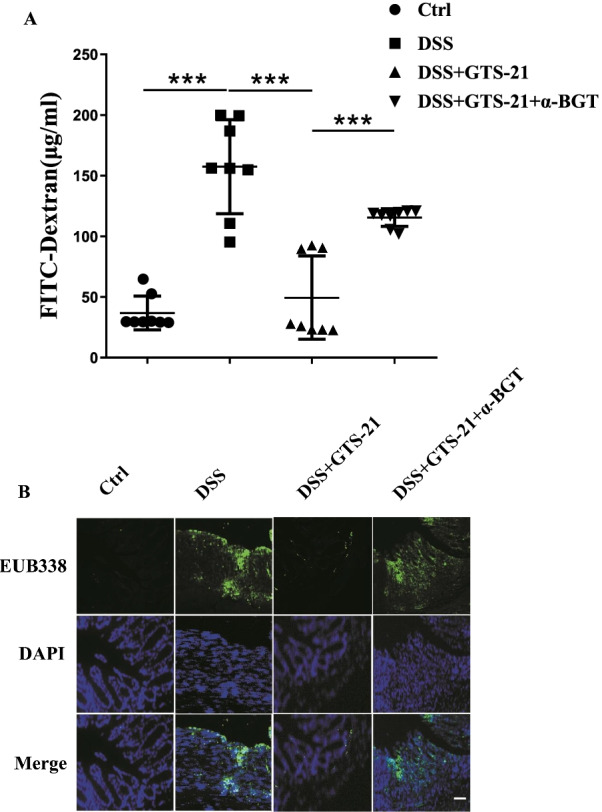
GTS-21 attenuates intestinal permeability and reduces intestinal bacterial translocation in DSS-induced colitis mice. **A** The gut mucosal permeability was evaluated by measuring the leakage of fluorescein isothiocyanate (FITC)–dextran from the intestinal pouch. ***p < 0.001. **B** Bacteria penetrating in DSS-induced colitis was determined by fluorescence in-situ hybridization (FISH) of EUB338 probe (green, EUB338 probe; blue, DAPI nuclear staining). Magnification, ×400. Scale bar, 100 nm

We evaluated the gut mucosal permeability by measuring the leakage of FITC-Dextran from the intestinal pouch. As shown in Fig. 2A, the amount of FITC-Dextran leakage increased in DSS group compared with Ctrl group (157.4 ± 13.7 μg/ml vs 36.8 ± 4.9 μg/ml). However, treatment with GTS-21 significantly reduced the amount of FITC-Dextran leakage in the DSS-induced mice (49.5 ± 12.1 μg/ml vs 157.4 ± 13.7 μg/ml). Furthermore, pre-treatment with α7nAChR antagonist α-BGT before GST-21 did not reduce the amount of leakage of FITC-Dextran in DSS-induced colitis mice (115.5 ± 2.6 μg/ml vs 49.5 ± 12.1 μg/ml).

To investigate bacteria invasion in DSS-induced colitis, we used fluorescent EUB338 probe technique to detect intestinal bacterial translocation. Immunostaining for bacteria showed that bacteria was virtually absent from the mucosal layer in Ctrl group. DSS-induced colitis mice experienced significantly increased intestinal bacterial translocation. Furthermore, GTS-21 treatment could attenuate intestinal bacterial penetration in DSS-induced colitis mice (Fig. 2B). These data indicated that GTS-21  had protective role in intestinal barrier function.

### α7nAChR agonist GTS-21 induces intestinal TJ proteins expressions and prevents TJs structure destruction

**Fig. 3:**
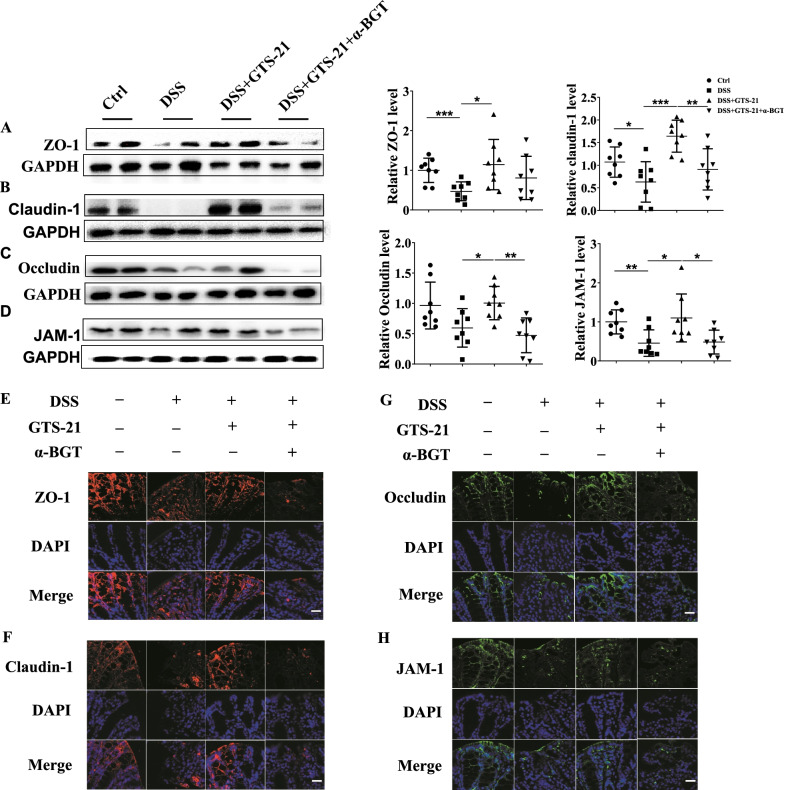
GTS-21 improves intestinal tight junction (TJ) proteins expressions in DSS-induced colitis. **A**–**D** Western blot analysis of mice colonic TJ proteins (ZO-1, claudin-1, occludin, JAM-1) expressions level in different groups. *p < 0.05; **p < 0.01, ***p < 0.001. **E**–**H** Representative immunofluorescence of ZO-1 and claudin-1 (red), occludin and JAM-1 (green) in the proximal colon of mice in Ctrl group (DAPI nuclear staining), DSS group, GTS-21 group and α-BGT pretreatment before GTS-21 group. Magnification, ×400. Scale bar, 100 nm

To determine the effect of GTS-21 on intestinal TJ proteins expressions, we analyzed the expressions of ZO-1, JAM-1, claudin-1 and occludin proteins by Western blot. As shown in Fig. 3A, B, D, the ZO-1, claudin-1 and JAM-1 proteins expressions in intestinal tissues of DSS-induced colitis mice significantly decreased, while occludin protein only showed a decreasing trend (Fig. 3C). GTS-21 treatment augmented the intestinal ZO-1, claudin-1, occludin and JAM-1 proteins expressions (Fig. 3A–D), while treatment with α7nAChR antagonist α-BGT abolished the role of GTS-21 (Fig. 3A–D).

We did immunofluorescence to determine the distribution of TJs. The results showed that the structure of ZO-1, claudin-1, occludin and JAM-1 were impaired in colonic tissues in DSS group. Treatment with GTS-21 prevented the destruction, while α7nAChR antagonist α-BGT counteracted the protective role of GTS-21 (Fig. 3E–H).

**Fig. 4:**
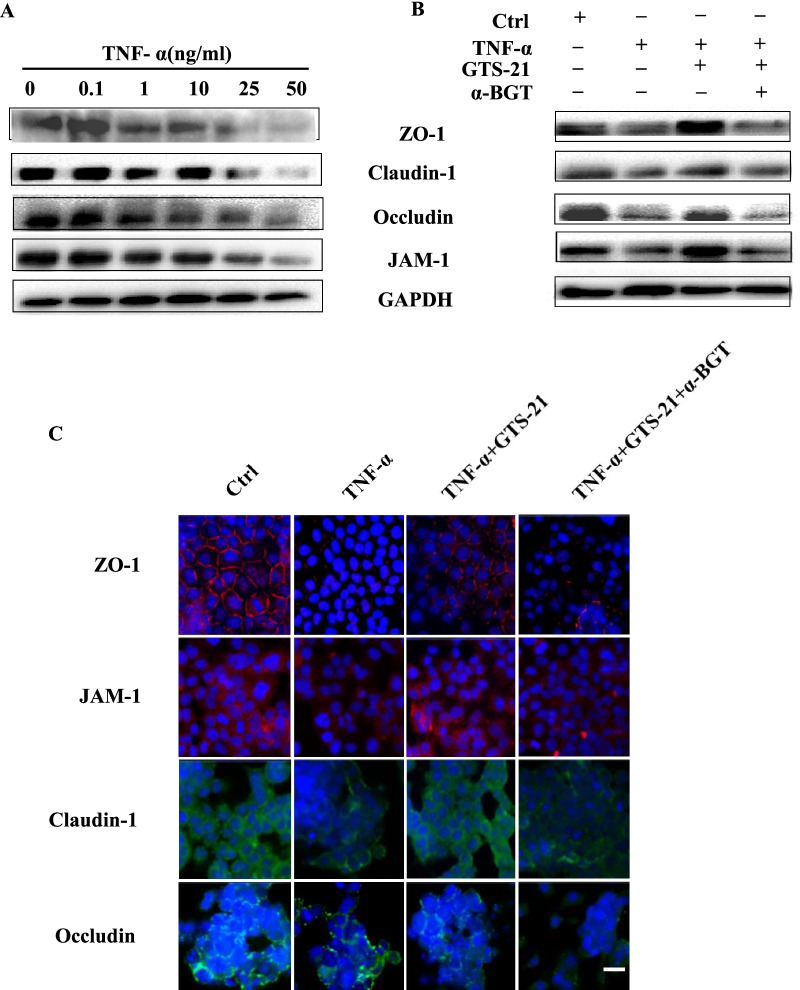
GTS-21 improves the tight junction (TJ) proteins expressions and distribution in Caco2 monolayers. **A** Western blot analysis of Caco2 cellular TJ proteins (ZO-1, claudin-1, occludin, JAM-1) expressions level in each group with stimulation of different concentrations of TNF-α for 24 h. **B** Western blot analysis of Caco2 cellular TJ proteins (ZO-1, claudin-1, occludin, JAM-1) expressions level in different groups. **C** Representative immunofluorescence of ZO-1 and JAM-1 (red), claudin-1 and occludin (green) in Caco-2 monolayers. (DAPI nuclear staining). Magnification, ×400. Scale bar, 100 nm

Subsequently, we chose Caco2 cell monolayer model to further investigate the effect of GTS-21 on the expressions and subcellular location of TJs proteins in vitro. As shown in Fig. 4A, TNF-α repressed the expressions of ZO-1, occludin and JAM-1 in concentration-dependent manner. However, treatment with GTS-21 upregulated ZO-1, claudin-1, occludin and JAM-1 expressions (Fig. 4B). Quantification of these results was shown in Additional file 1: Fig. S1. Meanwhile, TNF-α destroyed the structure of ZO-1, claudin-1, occludin and JAM-1 in Caco2 cell monolayer model. GTS-21 treatment alleviated the destruction, however, α7nAChR antagonist α-BGT reversed the protective role of GTS-21 (Fig. 4C).

### α7nAChR agonist GTS-21 attenuates intestinal epithelial TJs damage through the NF-қB pathways

**Fig. 5:**
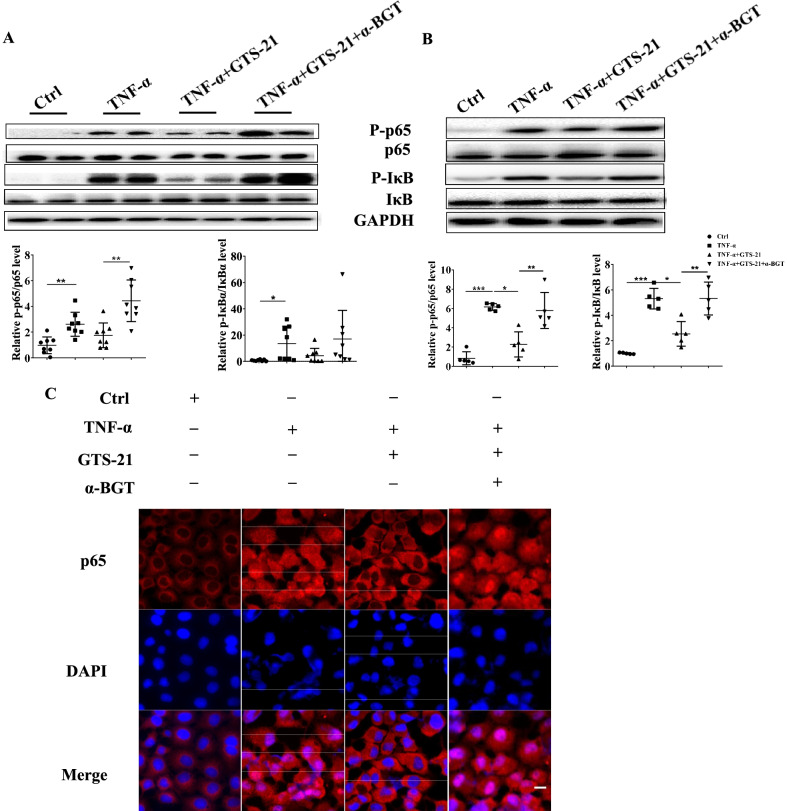
GTS-21 attenuates NF-қB activation in the intestinal tissues both in DSS-induced colitis and Caco2 monolayers. **A** Western blot analysis of NF-κB associated proteins (phosphorylated and non-phosphorylated IκBα, NF-қB p65) expressions level in colonic tissues, *p < 0.05, **p < 0.01. **B** Western blot analysis of NF-κB associated proteins (phosphorylated and non-phosphorylated IκBα, NF-қB p65) expressions level in Caco2 cells incubated with TNF-α for 30 min, *p < 0.05, **P < 0.01, ***p < 0.001. **C** The activation of NF-κB was detected by NF-κB nuclear translocation assays. Representative immunofluorescence of p65 (red) in Caco2 monolayers. (DAPI nuclear staining). Magnification, ×400. Scale bar, 100 nm

To explore the potential mechanism of GTS-21 in reducing intestinal epithelial TJs damage, we verified the effect of GTS-21 on NF-қB activation in colon tissues. As shown in Fig. 5A, the phosphorylation level of NF-κB p65 and IκBα proteins significantly increased in the DSS group. GTS-21 downregulated the phosphorylation level of p65 and IκBα proteins, while α-BGT administration mitigated the inhibitory effect of GTS-21. In vitro, we used Caco2 cell monolayer model to further elucidate this phenomenon. As shown in Fig. 5B, the phosphorylation level of p65 and IκBα proteins in Caco2 cells, which  significantly elevated when cells were treated with TNF-α,  decreased after GTS-21 intervention. Figure 5C showed that GTS-21 reduced nuclear translocation of NF-қB when Caco2 cells were stimulated by TNF-α and α-BGT administration partially blocked this effect.

## Discussion

The onset of DSS colitis could be restrained due to upregulation of Ach, which was non-selective agonist of nAChR, by vagal nerve stimulation using electrical stimulation (Meroni et al. 2021). As a homomeric receptor subtype, α7nAChR played an irreplaceable role as part of nAChR in CAP (Kalkman and Feuerbach 2016). It has been revealed that GTS-21 inhibited inflammatory cytokines secretion from peripheral blood immune cells (Engler et al. 2018). However, the influence of GTS-21 on intestinal barrier function was still not sure. In our study, we found that α7nAChR agonist GTS-21 ameliorated DSS-induced colitis, which might be due to reducing gut permeability, villus damage and inflammatory cell infiltration. Furthermore, we found that GTS-21 could attenuate enteric bacterial translocation caused by barrier function injury in DSS-induced colitis mice.

Paracellular pathway of epithelial layer is a prominent way for bacterial and related toxins translocation. TJ proteins interact with each other to form intestinal barrier and regulate paracellular pathway. In our study, we showed that the reason that GTS-21 decreased intestinal permeability might be upregulating expressions and remodeling the structure of ZO-1, claudin-1 and JAM-1 proteins in colon tissues in mice treated with DSS. However, GTS-21 could inhibit TNF-α, IL-1β and HMGB1 secretion from immune cells (Báez-Pagán et al. 2015). These inflammatory cytokines had detrimental influence on assembly and expressions of TJ proteins (Soyka et al. 2012; Chen et al. 2015). Although we have certified the existence of α7nAChR in intestinal epithelial cells and Caco2 cells (Additional file 2: Fig. S2), which was fundamental to GTS-21 treatment, the effects of GTS-21 on TJ proteins in DSS colitis might be indirect.

To further elucidate whether GTS-21 had protective role on barrier function directly, Caco2 cells was used as the model of intestinal epithelial cells layer. Considering the widely use and efficiency of TNF-α blockers in patients with IBD (D’Haens and van Deventer 2021), we chose TNF-α as stimulus to do research in vitro. It demonstrated that GTS-21 enhanced the expressions of ZO-1, claudin-1, occludin and JAM-1 in TNF-α treated Caco2 monolayer model. In addition, there are other isoforms of claudins expect for claudin-1. For instance, the expression of claudin-2, an important part of water channel in intestine (Rosenthal et al. 2010) and modulator of immune balance in intestine (Ahmad et al. 2014), was upregulated in IBD patients (Luettig et al. 2015). Now, the role that GTS-21 acts on claudin-2 is still unknown, which is worth to studying. Also, given the possibility that the effect of different cytokines made on TJ proteins may be distinct, additional model should be used to further certify possible mechanisms.

Recent studies have suggested that intestinal barrier damage and abnormal expressions of TJ proteins might be mediated by NF-қB activation (Ma et al. 2004). In the present study, we observed that α7nAChR agonist GTS-21 could decrease the phosphorylation level of IκBα and NF-қB P65 in DSS-induced colitis, while pre-treatment with α7nAChR antagonist α-BGT suppressed this influence. In TNF-α treated Caco2 monolayer model, the same phenomenon could be observed when synergistically used GTS-21 and TNF-α. There were binding sites for activated NF-қB in myosin light chain kinases (Al-Sadi et al. 2016), which were indispensable for relocation of TJ proteins and formation of scaffold (Jin and Blikslager 2020). Moreover, GTS-21 could decrease inflammatory cytokines secretion through inhibiting NF-қB pathway (Báez-Pagán et al. 2015). In all, GTS-21 could make effects on expressions and restructure of TJ proteins both directly and indirectly.

There are several limits in our study. Though vagal nerve stimulation had protective and therapeutic role in different experimental colitis models (Nunes et al. 2019), there seemed to be some obstacles in the way to get satisfied progress in clinical treatment, which might be due to the existence of CHRFAM7A that had ability to suppress CHRNA7 and uniquely expressed in human (Costantini et al. 2015). In vivo, we weren’t able to make the same circumstances to mimic the influence of GTS-21 on human. In vitro model, we didn’t study the expression of CHRFAM7A in Caco2 cells under GTS-21 treatment due to cross-reactivity between the two antibodies. Although we have made sure that GTS-21 could restore intestinal barrier directly on the existence of CHRFAM7A, adding to that it was capable of decreasing inflammatory tone in peripheral blood immune cells which also expressed this gene, CHRFAM7A-transgenic mice should still be utilized as a preclinical model to further investigate the relationship and interaction between immune cells and epithelial cells under treatment of GTS-21, which might be helpful to fill the gaps and translate into clinical application.

## Conclusions

In conclusion, our results indicated that α7nAChR agonist GTS-21 alleviated intestinal inflammatory damage, which might be related to decreasing the intestinal mucosal permeability and increasing expressions of TJs proteins. GTS-21 inhibited NF-қB activation through α7nAChR. Our data provided new insight into the role of α7nAChR agonist GTS-21 in attenuating intestinal barrier damage, which might be used as barrier-protection drug in the future.

## Supplementary Information

**Additional file 1: Figure S1.** Quantification of Western blot results. (A) Quantification of Western blot results in Fig. 4A, *p < 0.05, **p < 0.01.vs 0 alone. (B) Quantification of Western blot results in Fig. 4B, *p < 0.05, **p < 0.01, ***p < 0.001.

**Additional file 2: Figure S2.** The expression of α7nAChR in Caco2 cells and intestinal epithelial cells. (A) Representative immunofluorescence image of intestinal epithelial cells stained with α7nAChR (red) and co-labeled with DAPI. (B) Western blot analysis of α7nAChR expression in Caco2 cells and representative immunofluorescence image of Caco2 cells stained with α7nAChR (red) and co-labeled with DAPI. Scale bar, 50 μm.

## Data Availability

The data used during the current study are available from the corresponding author on reasonable request.

## Abbreviations

α7nAChR: α7 nicotinic acetylcholine receptor
GTS-21: 3-(2,4 Dimethoxy-benzylidene)-anabaseine dihydrochloride
DAI: Disease activity index
HAI: Histological activity index
FITC: Fluorescein isothiocyanate
TJ: Tight junction
IBD: Inflammatory bowel disease
CD: Crohn’s disease
UC: Ulcerative colitis
Ach: Acetylcholine
CAP: Cholinergic anti-inflammatory pathway
TNF-α: Tumor necrosis factor alpha
HMGB1: High-mobility group box1
FISH: Fluorescence in-situ hybridization
NF-κB: Nuclear factor kappa-light-chain-enhancer of activated B cells
IκB: Inhibitor of NF-κB, alpha
CHRNA7: Cholinergic Receptor, Nicotinic, Alpha 7
CHRFAM7A: Cholinergic Receptor Family with Sequence Similarity 7A
