# Supplementary figures and images for: α7 nicotinic acetylcholine receptor agonist GTS-21 attenuates DSS-induced intestinal colitis by improving intestinal mucosal barrier function
Source: Mol Med. 2022 Jun 3;28:59. doi: 10.1186/s10020-022-00485-6 (PMC9164421; doi:10.1186/s10020-022-00485-6)

**Figure S1**

**A**

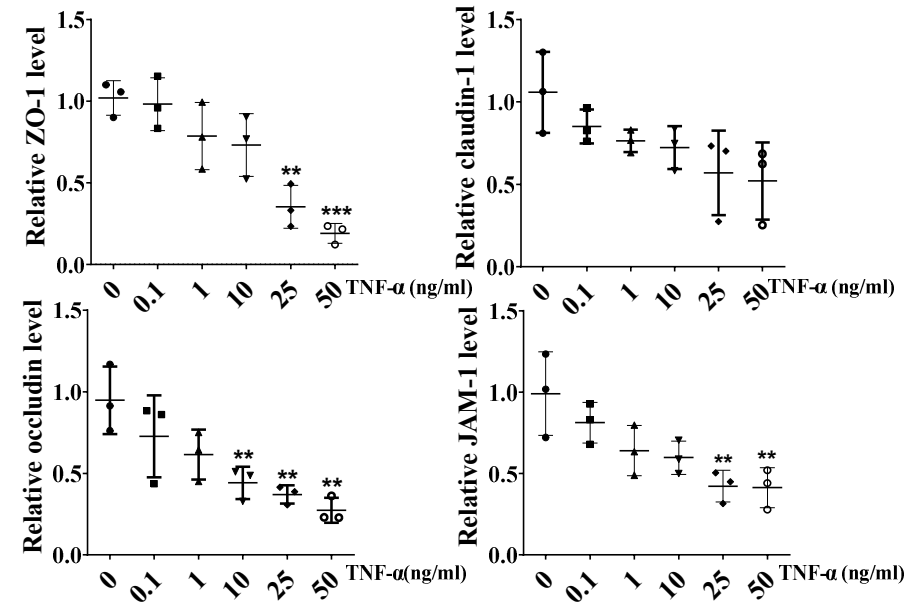

**B**

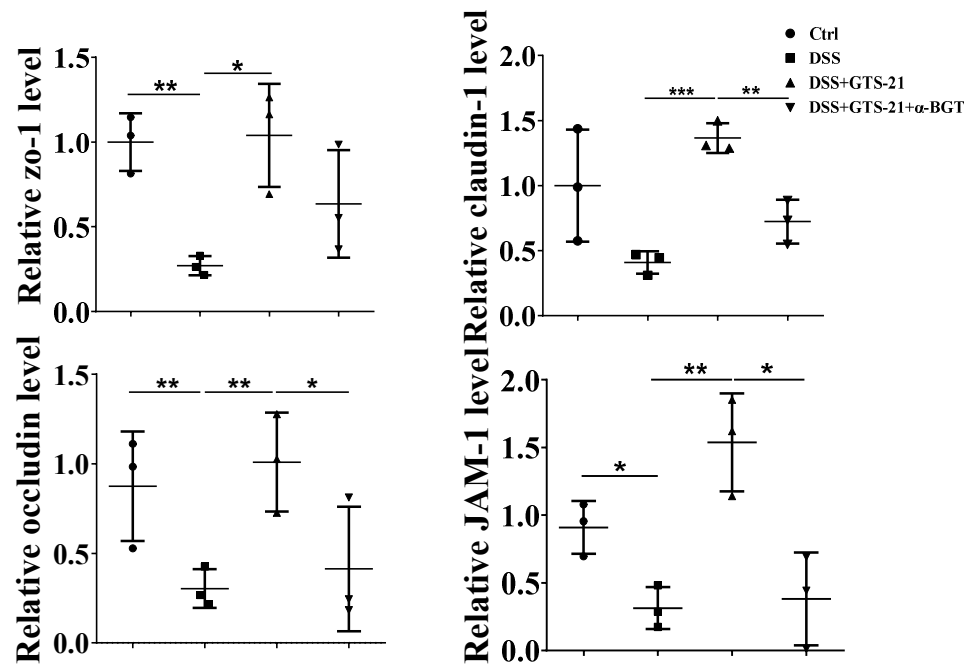

Supplement: Supplementary file 1 — Additional file 1: Figure S1. Quantification of Western blot results. (A) Quantification of Western blot results in Fig. 4A, *p < 0.05, **p < 0.01.vs 0 alone. (B) Quantification of Western blot results in Fig. 4B, *p < 0.05, **p < 0.01, ***p < 0.001. [file 10020_2022_485_MOESM1_ESM.pdf]

**Figure S2**

**A**

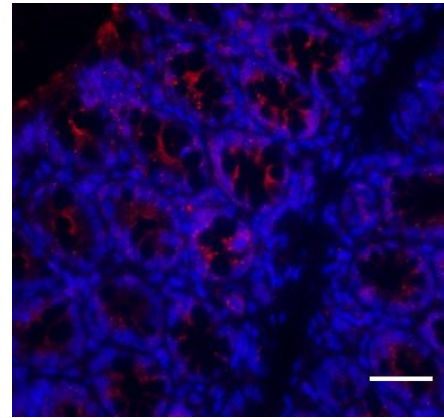

**B**

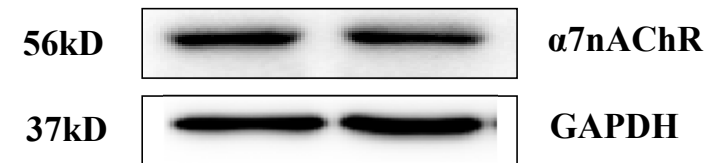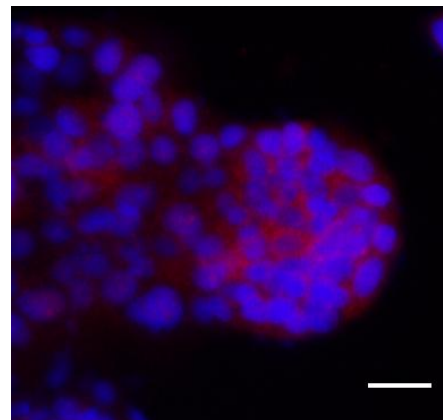

Supplement: Supplementary file 2 — Additional file 2: Figure S2. The expression of α7nAChR in Caco2 cells and intestinal epithelial cells. (A) Representative immunofluorescence image of intestinal epithelial cells stained with α7nAChR (red) and co-labeled with DAPI. (B) Western blot analysis of α7nAChR expression in Caco2 cells and representative immunofluorescence image of Caco2 cells stained with α7nAChR (red) and co-labeled with DAPI. Scale bar, 50 μm. [file 10020_2022_485_MOESM2_ESM.pdf]
